# Léiomyome cutané: une autre cause d'ulcère de jambe

**DOI:** 10.11604/pamj.2015.22.134.7959

**Published:** 2015-10-13

**Authors:** Redouane Ouakrim, Mustapha Mahfoud

**Affiliations:** 1 Service de Chirurgie Orthopédique, CHU Ibn Sina, Université V, Souissi, Rabat, Maroc

**Keywords:** Léiomyome, cutané- ulcère, tumeur, leiomyoma, cutaneous - ulcers, tumor

## Image en medicine

Le léiomyome est une tumeur bénigne décrite initialement par Virchow en 1854, il représente 3,8% de toutes les tumeurs de tissu mou. Le léiomyome cutané est souvent multifocal et très douloureux au toucher et au froid. Les membres, le tronc et les régions latéro-faciales et latéro-cervicales sont les sites de prédilection. Elles peuvent rentrer dans le cadre de syndromes complexes (syndrome de Gardner), associées à des endocrinopathies, ou d'autres tumeurs bénignes telles que les lipomes. Le diagnostic est histologique. Le traitement est chirurgical et est difficile à mettre en œuvre, vu le nombre important de lésions que l'on peut rencontrer. Après traitement chirurgical, la récidive survient, dans la majorité des cas au même endroit. Une marge chirurgicale de 1 cm est préconisée pour l'exérèse de ces formes récidivantes. Nous rapportons le cas d'un patient de 28 ans, sans antécédents, avait consulté pour une tumeur de 3cm de diamètre, la peau en regard était érythémateuse et dépilée avec une ulcération centrale. L’étude histologique couplée à l'immunohistochimie a permis de retenir le diagnostic d'un léiomyome cutané pilaire. Une exérèse chirurgicale suivie d'une greffe cutanée était réalisée. Le patient n'a pas fait de récidive avec un recul de 2 ans.

**Figure 1:**
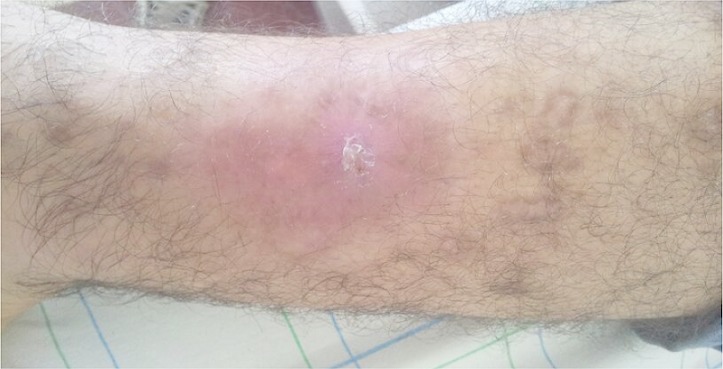
Ulcère chronique de jambe révélant un léiomyome cutané

